# Supplementary material for: Emergence and control of photonic band structure in stacked OLED microcavities
Source: Nat Commun. 2021 Oct 20;12:6111. doi: 10.1038/s41467-021-26440-3 (PMC8528838; doi:10.1038/s41467-021-26440-3)
Supplement: Supplementary file 4 — Supplementary Data 1 [file 41467_2021_26440_MOESM4_ESM.zip › OLED Simulation v2-1/OLED Simulation/Materials Data/Materials Database/rii-database-2019-02-11.zip!/database/info/main/Fe2O3.html]

# Iron sesquioxide, Fe_2_O_3_

## Other names

- Iron(III) oxide
- Diiron trioxide
- Iron oxide
- Ferric oxide
- Ferric iron
- Red iron oxide
- Rouge
- Colcothar
- Rust
- Ochre

## Minerals

- Hematite (haematite)
- Maghemite

## External links

- Iron(III) oxide - Wikipedia
- Diiron trioxide - WebElements
- Hematite - Wikipedia
- Maghemite - Wikipedia
- Rust - Wikipedia
- Ochre - Wikipedia
